# Validation of the Dutch Aging Perceptions Questionnaire and development of a short version

**DOI:** 10.1186/s12955-015-0248-y

**Published:** 2015-05-12

**Authors:** Anne Slotman, Jane M Cramm, Anna P Nieboer

**Affiliations:** Institute of Health Policy and Management, Erasmus University of Rotterdam, Rotterdam, the Netherlands

**Keywords:** Aging perceptions, APQ, B-APQ, Validity, Reliability

## Abstract

**Background:**

Perceptions of aging have been found to independently contribute to various aspects of health and wellbeing in old age. Since valid and reliable perceptions of aging instruments are unavailable in Dutch, these associations have not yet been tested in the Netherlands. This study examined the reliability and construct validity of the Dutch-language version of the 7-dimension Aging Perceptions Questionnaire (APQ). Furthermore, in order to decrease the response burden, while retaining the APQ’s original factor structure, a short version of the APQ (APQ-S) was developed as an alternative to the 5-dimension Brief APQ (B-APQ).

**Methods:**

A Dutch translated version of the APQ was administered to a large sample of community-dwelling elders in the Netherlands, aged 70 to 99 (*n* = 1280), alongside measures of wellbeing and physical functioning.

**Results:**

Confirmatory factor analyses confirmed the multidimensionality of the APQ. APQ scales were found to have good reliability and acceptable construct validity, yet several areas of localized strain were detected. These areas were addressed during item reduction, resulting in the 21-item APQ-S with an acceptable reliability and validity and a better overall model fit. While several notable differences were found, APQ-S results were largely comparable to that of the 5-dimension B-APQ.

**Conclusion:**

With its multidimensional nature and acceptable psychometric properties, the Dutch language version of the APQ may prove to be an invaluable instrument to assess the seven perceptions of aging dimensions among older populations for geriatric research. However, use of a shortened version is advised, as these are less labor intensive and areas of localized strain are addressed. The choice between the APQ-S and the B-APQ should be based on theoretical and practical considerations concerning the dimensional structure most suitable for the study.

**Electronic supplementary material:**

The online version of this article (doi:10.1186/s12955-015-0248-y) contains supplementary material, which is available to authorized users.

## Background

While aging is regularly described as a debilitating process characterized by an accumulation of functional limitations, due to gradual physical and/or cognitive decline, it is not always experienced as such. On the contrary, literature suggests that a majority of elderly people have positive perceptions of aging [1,2] and/or do not even label themselves as ‘old’ [1]. Several studies underscore the merits of having a positive instead of a negative view on the aging process; those who were generally optimistic about aging engaged in more health-promoting behavior [3], reported feeling less lonely [4] and depressed [5], had better functional health [2], and even had lower mortality rates [6,7].

Yet, despite its importance to geriatric research, only a limited number of valid and reliable measurement instruments have been developed to explore perceptions of aging. One of these instruments is the 32-item Aging Perceptions Questionnaire (APQ) [8]. Recognizing the complex and multi-faceted nature of the aging process, the APQ adopts a multi-dimensional approach to quantitatively assess people’s aging experience. Following Leventhal’s self-regulation model [9], the APQ theorizes that people form representations of aging in order to make sense of and react to the aging process. Underlying these representations is a series of dimensions, which correspond to the (sub)scales of the APQ [8]:

- *Timeline*: the awareness of aging and the experience of the process over time. This dimension is divided into two sub-dimensions: *timeline chronic*, an awareness of aging that is chronic in nature (e.g., ‘I always classify myself as old’), and *timeline cyclical*, an awareness of aging that comes and goes in waves (e.g., ‘I go through phases of feeling old’).
- *Consequence*: beliefs about the impact of aging on various life domains. This dimension is comprised of two sub-dimensions: *consequences positive* (e.g., ‘As I get older I get wiser’) and *consequences negative* (e.g., ‘Getting older makes me less independent’).
- *Emotional representations*: the emotional response generated by aging (e.g., ‘I get depressed when I think about getting older’).
- *Control*: beliefs about the extent to which one can manage different aspects of aging. Two sub-dimensions are present: *control positive*, control beliefs concerning positive experiences (e.g., ‘Whether I continue living life to the full depends on me’) and *control negative*, control beliefs concerning negative experiences (e.g., ‘How mobile I am in later life is not up to me’).

Originally developed in Ireland, the APQ has been applied to other samples of community-dwelling elders in Ireland [10,11] and Australia [12]. Furthermore, it has since been translated to Brazilian-Portuguese [13] and translated and validated for use in France [14]. To date, the APQ has not been translated to Dutch and administered to a sample of community-dwelling elders in the Netherlands.

Moreover, the APQ is a relatively lengthy instrument. As the burden of responding and, in turn, the rate of non-response generally increases with the length of the questionnaire [15], the length of the APQ could be problematic, especially among (frail) older people for whom answering questionnaires often carries more difficulties. Development of a shortened version of the APQ could solve this problem. While recently a brief version of the APQ, the B-APQ [16], has been developed, this version may not be the best alternative for all studies, as in the B-APQ the original APQ dimensions are modified by conjoining the consequence and control negative dimensions and excluding timeline cyclical. Hence, for those who wish to investigate each aspect of perceptions of aging, as originally theorized in the APQ, a shortened questionnaire which retains the original 7-dimension structure may be more suitable.

The current paper aims to contribute to further development and validation of the APQ by (a) examining its psychometric properties in a large sample of community-dwelling elders in the Netherlands and (b) reducing its number of items while maintaining validity and reliability and, in specific, its original 7-dimension structure.

## Methods

### Participants and procedure

The Dutch-language APQ was part of a larger questionnaire administered among elderly community-dwelling people in Rotterdam, the Netherlands (see study protocol [17]). Participants were randomly sampled, stratified by age group (70 – 74, 75 – 79, 80 – 84, and ≥ 85) and neighborhood, through the Rotterdam municipality register. The number of participants per neighborhood was proportionally weighted to the population ratio of the district. Only one person per address was allowed to participate. Participants were sent a written questionnaire alongside an invitation and self-addressed envelope. Two reminders were sent in case of non-response. In total, 2890 people were approached. Sixty-seven respondents were excluded prior to the study, since they resided in nursing-homes or were hospitalized. Another 25 respondents could not participate due to serious medical issues (i.e., dementia) or death. Of the remaining 2798 respondents, 1280 returned a filled-in questionnaire, achieving a response rate of 46%.

### Measures

#### Aging Perceptions Questionnaire

The APQ consists of 32 items, comprising seven (sub)scales, i.e., timeline chronic, timeline cyclical, consequence positive, consequence negative, emotional representations, control positive, and control negative (see Table 1 for the list of items). Answers are provided on a 5-point scale, ranging from 1 ‘strongly disagree’ to 5 ‘strongly agree’. The response scale of the control negative dimension is reversed (1 ‘strongly agree’ to 5 ‘strongly disagree’) and thus has to be recoded for further analyses. The original APQ items were translated into Dutch by a professional English translator (see Additional file 1 for the Dutch translation of the APQ).

**Table 1:** **Item characteristics and standardized factor loadings of the APQ**

**Items**	**Missing (%)**	***M***	**SD**	**Med**	**λ**
*Timeline chronic*					
1. I am conscious of getting older all of the time	2.00	4.03	.84	4.00	.60
**2. I am always aware of my age**	**2.70**	**3.54**	**1.06**	**4.00**	**.76**
**3. I always classify myself as old**	**3.30**	**2.92**	**1.14**	**3.00**	**.75**
**4. I am always aware of the fact that I am getting older**	**3.00**	**3.59**	**1.02**	**4.00**	**.78**
5. I feel my age in everything that I do	3.50	3.03	1.14	3.00	.75
*Timeline cyclical*					
**27. I go through cycles in which my experience of ageing gets better and worse**	**6.10**	**2.95**	**1.04**	**3.00**	**.71**
28. My awareness of getting older comes and goes in cycles	7.10	3.10	1.03	3.00	.62
**30. I go through phases of feeling old**	**5.60**	**2.88**	**1.12**	**3.00**	**.76**
**31. My awareness of getting older changes a great deal from day to day**	**6.30**	**2.69**	**1.06**	**3.00**	**.72**
32. I go through phases of viewing myself as being old	5.90	2.89	1.14	3.00	.76
*Consequence positive*					
**6. As I get older I get wiser**	**4.40**	**3.21**	**1.00**	**3.00**	**.74**
**7. As I get older I continue to grow as a person**	**6.20**	**3.15**	**.94**	**3.00**	**.83**
**8. As I get older I appreciate things more**	**3.60**	**3.82**	**.85**	**4.00**	**.55**
*Consequence negative*					
**16. Getting older restricts the things that I can do**	**5.50**	**3.56**	**.94**	**4.00**	**.67**
17. Getting older makes me less independent	7.20	3.18	.99	3.00	.50
**18. Getting older makes everything a lot harder for me**	**6.50**	**3.09**	**1.04**	**3.00**	**.85**
**19. As I get older I can take part in fewer activities**	**4.70**	**3.49**	**.99**	**4.00**	**.71**
20. As I get older I do not cope as well with problems that arise	5.30	3.17	1.03	3.00	.69
*Emotional representations*					
9. I get depressed when I think about how ageing might affect the things that I can do	3.40	2.90	1.16	3.00	.70
13. I get depressed when I think about the effect that getting older might have on my social life	5.60	2.67	1.06	3.00	.78
**25. I get depressed when I think about getting older**	**6.50**	**2.56**	**1.06**	**2.00**	**.85**
**26. I worry about the effects that getting older may have on my relationships with others**	**6.20**	**2.67**	**1.02**	**3.00**	**.74**
**29. I feel angry when I think about getting older**	**5.50**	**2.15**	**.97**	**2.00**	**.63**
*Control positive*					
**10. The quality of my social life in later years depends on me**	**3.60**	**3.82**	**.87**	**4.00**	**.85**
11. The quality of my relationships with others in later life depends on me	3.40	3.84	.83	4.00	.84
**12. Whether I continue living life to the full depends on me**	**2.70**	**3.57**	**.97**	**4.00**	**.61**
14. As I get older there is much I can do to maintain my independence	5.00	3.82	.82	4.00	.41
**15. Whether getting older has positive sides to it depends on me**	**5.50**	**3.75**	**.81**	**4.00**	**.49**
*Control negative*					
**21. Slowing down with age is not something I can control**	**4.70**	**2.27**	**.87**	**2.00**	**.62**
**22. How mobile I am in later life is not up to me**	**5.20**	**2.40**	**.93**	**2.00**	**.73**
**23. I have no control over whether I lose vitality or zest for life as I age**	**4.90**	**2.62**	**1.03**	**2.00**	**.77**
24. I have no control over the effects which getting older has on my social life	5.90	2.98	1.03	3.00	.59

*Note.* Items 21 to 24 are reverse coded. Items in bold are the items included in the short version of the APQ (APQ-S).

#### Wellbeing

Wellbeing was measured by the 15-item version of the Social Production Function Instrument for the Level of wellbeing (SPF-IL) [18]. The SPF-IL assesses whether a person’s need for affection, status, behavioral confirmation, comfort, and stimulation is fulfilled. Response categories are provided on a 4-point scale with higher scores being indicative of better experienced wellbeing. Previous studies verify the reliability of the SPF-IL as an instrument to assess wellbeing among elderly populations [19,20]. Cronbach’s alpha of the SPF-IL in this study was .86, indicating good internal consistency. An overall mean score was calculated to facilitate further analyses.

#### Physical functioning

The Physical Functioning scale of the Tilburg Frailty Index (TFI-PF) [21] was used to assess the physical functioning of the respondents. The TFI-PF contains 8 yes/no questions, which provide information about experienced difficulties with daily activities and one’s ability to be active. Earlier studies support the reliability and validity of the TFI [21-23]. A Cronbach’s alpha of .75 was found in this study, indicating acceptable reliability. The items were reverse coded and summed to create an overall score, with higher scores being indicative of better physical functioning.

#### Socio-demographic variables

Age, gender, ethnicity, educational level, marital status, and monthly income were taken into account as control variables. The variable monthly income was created by dividing the monthly household income by the number of household members. To facilitate model estimation by reducing the number of parameters within the model, gender, ethnicity, educational level, marital status, and monthly income were dichotomized, with male, Dutch, more than elementary schooling, married/living together, and monthly income above €1000 as reference categories, respectively. Descriptive statistics are available in Table 2.

**Table 2:** **Descriptive statistics for variables used in the regression analyses**

	***M*** **(SD)/%**	**Missing (%)**
Wellbeing (1 - 4)	2.74 (.47)	3.10
Physical functioning (0 - 8)	5.75 (2.10)	3.10
Age (70 - 99)	78.59 (6.17)	0
Gender (%)		
Female	57.60	0
Income (%)		
≤ € 1000	8.80	18.00
Education (%)		
≤ Elementary	33.00	4.00
Marital status (%)		
Single/widow	59.30	2.00
Ethnic/migrant group (%)		
Non-Dutch	15.20	0

*Note.* The displayed means and standard deviations or proportions are calculated after missing data handling.

### Analyses

Data analysis was performed, using the following sequence of steps:

1. Item means, standard deviations, and medians were calculated in combination with visual inspection of histograms and Q-Q plots in order to detect floor or ceiling effects. Furthermore, the data was screened for univariate outliers. Item non-response rates were inspected to find questions that might have been misunderstood. A percentage of missing values under 10% was seen as acceptable. This information was taken into account during the item reduction phase of the analysis.
2. To examine the factor structure of the APQ, a Confirmatory Factor Analysis (CFA) was performed, using Structural Equation Modeling (SEM) in Mplus 7.0 [24]. SEM allows for constructing and validating measurement models and provides multiple model fit statistics and modification indices, which facilitates item reduction and evaluation and comparison of the original and shortened APQ. Seven latent variables were specified, corresponding to the seven (sub)scales of the APQ. The measurement model contained no double loadings or correlated measurement errors. Latent variables were allowed to correlate. Goodness of fit was evaluated using Chi Square test statistics in combination with the Standardized Root Mean Square Residual (SRMR), Root Mean Square Error of Approximation (RMSEA), and the Comparative Fit Index (CFI). In accordance with Hu and Bentler [25], acceptable model fit was defined by the following criteria: SRMR ≤ .08; RMSEA ≤ .06; and CFI ≥ .90. Furthermore, a small and non-significant Chi Square indicates exact model fit. However, due to its sensitivity to large sample size, the Chi Square statistics must be interpreted with caution. Full Information Maximum Likelihood (FIML) was used to account for missing values, as methodologists generally regard this method to be best suited for handling missing data in most CFA and SEM applications [26-28]. Participants who responded to fewer than 16 variables of the APQ were excluded from analyses (*n* = 60).
3. Cronbach’s alpha was calculated in SPSS 19.0 to assess the internal consistency of the APQ subscales and compare it to the findings of Barker and colleagues [8]. Also, subscale distributions were examined for floor or ceiling effects by calculating subscale means and standard deviations and visually inspecting subscale frequency distributions.
4. In order to determine construct validity of the APQ, correlations between the subscales within each instrument were calculated and compared to the correlations found in the study by Barker and colleagues [8]. Inter-factor correlations were not allowed to exceed .80, as this may be indicative of a lack of discriminant validity [28].
5. Following Barker and colleagues [8], construct validity was further evaluated by performing stepwise regression analyses. Structural models were specified, using Mplus 7.0 in which the APQ dimensions were regressed on the wellbeing or physical functioning measures, i.e., the SPF-IL and TFI-PF. Socio-demographic variables were entered in the first step to better assess the contribution of the APQ dimensions while controlling for more traditional measures [8]. Only those that significantly correlated with the two outcome measures, as a result of preliminary bivariate correlations, were considered. Mean substitution was used to handle missing values on the socio-demographic variables (educational level (4%), marital status (2%), and monthly income (18%)) and the two outcome measures. Respondents who did not respond to at least 10 items of the SPF-IL (*n* = 41) or 6 items of the TFI-PF (*n* = 57) were excluded from the respective analyses. Comparable results were found when using listwise deletion for the socio-demographic variables.
6. Item reduction was performed in order to create a shorter version of the APQ, referred to as APQ-S in the following sections. Item exclusion was based on several criteria. First, exclusion was based on the presence of localized strain (i.e., areas of ill fit within the model due to, for example, correlated measurement errors [28]) as evidenced by large Modification Indices (MI) (>10) and Expected Parameter Change (EPC) values (> .20). Items with large error covariances or cross-loadings were opted for removal. Second, indicators with weak standardized factor loadings were selected (λ < .40) [29]. Furthermore, item response rates and floor or ceiling effects were taken into account. Finally, the Cronbach’s alpha of each subscale was not allowed to drop below .70. The final decision to exclude an item was also guided by theoretical considerations to avoid misrepresentation of the factors’ underlying constructs due to item reduction. The aim was to reduce the number of items to a minimum of three per dimension.
7. In order to examine whether the dimensions remained comparable after item removal, correlations were calculated between the APQ and APQ-S subscales. A positive correlation above .90 was seen as an indicator of adequate subscale similarity. Comparability of the APQ and APQ-S subscales was further based upon subscale means and standard deviations.
8. To assess whether the reliability and validity were maintained in the APQ-S, Cronbach’s alphas and inter-factor correlations were calculated and stepwise regression analyses performed. Subsequent results were contrasted with the APQ. An additional validity and reliability test of the APQ-S was conducted by examining common sense differences in APQ-S dimensions for each socio-demographic variable. For example, correlations were calculated between the APQ-S dimensions and age. As one can expect older age to be correlated with a more chronic awareness of aging, the presence of such a relation may further support the validity of the measure.
9. The APQ-S was compared to the B-APQ [16]. Comparison was based upon item-factor differences, model fit statistics, inter-factor and inter-instrument correlations, and Cronbach’s alphas.

## Results

### Data screening

The data were investigated for normality in terms of skewness and kurtosis and for univariate outliers. Several items were found to be skewed (see Table 1 for item means, standard deviations, and medians). This was taken into account during the item reduction analysis. Non-response was evenly distributed across items with rates of non-response between 2.0% and 7.2%. Approximately 77% of the participants responded to all the items.

### Confirmatory factor analysis

Based on the article by Barker and colleagues [8], a 7-factor measurement model was specified with the 32 indicators loading onto their corresponding latent dimensions, i.e., timeline chronic, timeline cyclical, consequence positive, consequence negative, control positive, control negative, and emotional representations. Since the assumption of normality was violated for multiple indicators, the sample variance-covariance matrix was analyzed, using maximum likelihood estimation with standard errors and a mean-adjusted Chi Square test statistic that are robust to non-normality (MLR) [24,28,30] (data available upon request).

Model fit statistics revealed a large and significant Chi Square (*χ*^2^(443) = 2043.91, *p* < .001), disproving the assumption of absolute model fit. Furthermore, the CFI statistic was slightly below the pre-set cut-off point of .90 (CFI = .88). However, SRMR and RMSEA indicated good fit (SRMR = .06; RMSEA = .05). Completely standardized parameter estimates of the measurement model are presented in Table 1. Factor loadings were coherent with previous findings, as all freely estimated unstandardized parameters were significantly related to its respective latent dimension (*p* < .001) and had standardized factor loadings above .40 (see Table 1). Yet, inspection of modification indices revealed several areas of localized strain, with multiple strong error covariances and cross-loadings, supporting the need for further model respecification.

### Reliability and subscale distributions

As can be seen in Table 3, Cronbach’s alpha of the APQ dimensions ranged from .74 to .86, indicating either acceptable (α ≥ .70) or good (α ≥ .80) reliability of the dimensions [31]. Compared to the Cronbach’s alphas found in the study by Barker and colleagues [8], the consequence positive dimension was notably more reliable in the current study. There were no large differences in subscales means across studies [8]. Finally, APQ subscales had no obvious floor or ceiling effects, as can be seen by the low percentage of respondents having a minimum or maximum subscale score.

**Table 3:** **APQ and APQ-S inter-factor and inter-instrument correlations, Cronbach’s alphas, and subscale descriptives**

**APQ Dimensions**	**1.**	**2.**	**3.**	**4.**	**5.**	**6.**	**7.**	***M*** **(SD)**	**% Min**	**% Max**	**α**
*1. Timeline chronic*											
APQ (2007)	-							2.90 (.87)	1.80	.80	.86
APQ	-							3.42 (.83)	2.70	.80	.85
APQ-S	-							3.35 (.92)	2.00	5.50	.81
*2. Timeline cyclical*											
APQ (2007)	.50^***^	-						2.70 (.82)	2.40	.20	.89
APQ	.49^***^	-						2.90 (.84)	.60	3.80	.84
APQ-S	.44^***^	-						2.84 (.88)	4.20	1.00	.76
*3. Consequence positive*											
APQ (2007)	-.07^***^	-.08^***^	-					3.70 (.59)	.20	2.60	.64
APQ	.23^***^	.11^***^	-					3.40 (.76)	3.50	.80	.74
APQ-S	.21^***^	.09^**^	-					3.40 (.76)	1.30	3.30	.74
*4. Consequence negative*											
APQ (2007)	.55^***^	.42^***^	-.09^***^	-				3.40 (.74)	.20	1.80	.80
APQ	.55^***^	.57^***^	.15^***^	-				3.30 (.76)	.80	1.90	.81
APQ-S	.50^***^	.54^***^	.12^***^	-				3.38 (.83)	1.50	4.10	.79
*5. Emotional representations*											
APQ (2007)	.53^***^	.65^***^	-.14^***^	.44^***^	-			2.40 (.74)	2.80	.10	.74
APQ	.44^***^	.71^***^	.10^**^	.56^***^	-			2.59 (.84)	1.30	3.30	.86
APQ-S	.33^***^	.66^***^	.07^*^	.46^***^	-			2.46 (.85)	7.00	1.20	.79
*6. Control positive*											
APQ (2007)	-.34^***^	-.24^***^	.28^***^	-.32^***^	-.37^***^	-		3.80 (.54)	.20	2.20	.80
APQ	.06	-.06^*^	.28^***^	.03	-.06^*^	-		3.76 (.64)	2.60	.90	.78
APQ-S	.04	-.06^*^	.27^***^	.01	-.06	-		3.71 (.70)	.70	5.40	.69
*7. Control negative*											
APQ (2007)	-.41^***^	-.32^***^	.06^**^	-.53^***^	-.38^***^	.26^***^	-	2.65 (.71)	1.60	.20	.73
APQ	-.44^***^	-.38^***^	-.15^***^	-.55^***^	-.44^***^	-.08^**^	-	2.56 (.74)	1.00	3.40	.76
APQ-S	-.37^***^	-.32^***^	-.17^***^	-.47^***^	-.33^***^	-.12^***^	-	2.43 (.77)	6.60	1.10	.76
*APQ & APQ-S*	.96^***^	.96^***^	1.00^***^	.94^***^	.94^***^	.95^***^	.95^***^	-			-

*Note.* ^*^ *p* ≤ .05; ^**^ *p* ≤ .01; ^***^ *p* ≤ .001. APQ (2007) results were found in the study by Barker and colleagues [8].

### Construct validity

#### Inter-factor correlations

Inter-factor correlations in Table 3 reveal significant relations between APQ dimensions. For example, those who ascribed more negative consequences to the aging process also reported more negative emotional responses to aging. The strongest correlation was found between timeline cyclical and emotional representations, indicating that those harboring more negative emotions towards aging also reported more variations in their experience of the process. Yet, this correlation did not exceed the critical point of .80 after which discriminant validity of the scale can be called into question [28].

Several noteworthy differences were found when comparing APQ inter-factor correlations across studies. Most remarkable was the inter-factor correlations for the consequence positive dimension that were, for all but the control positive dimension, in the opposite direction. For example, while in the study by Barker and colleagues [8] consequence positive was negatively correlated with consequence negative and emotional representations, in the current study these correlations were positive. Second, compared to the study by Barker and colleagues [8], inter-factor correlations for control positive were markedly weaker. Furthermore, a negative instead of positive correlation between control positive and control negative was found.

#### Stepwise regression analyses

Regression analyses were performed to further evaluate the construct validity of the APQ. As can be seen in Table 4, the socio-demographic variables explained 2% of the variance in wellbeing scores. Being of older age, widowed/single, or having a low educational level was significantly related to worse wellbeing. By including the APQ dimensions in model II, the model explained another 29% of the variance in wellbeing scores. Significant APQ dimensions were timeline cyclical and the consequence and control dimensions, with consequence negative having the strongest coefficient. While having a more cyclical perception of aging and attributing more negative consequences to the aging process was related to worse wellbeing, attributing more positive consequences to aging and perceiving more control over positive events was related to better wellbeing. Perceiving to be more in control over negative experiences associated with aging (e.g., becoming less independent) was related to less wellbeing.

**Table 4:** **Stepwise regression analyses explaining wellbeing (** ***n*** **= 1197) and physical functioning scores (** ***n*** **= 1189)**

	**Wellbeing**			**Physical functioning**		
**Variables**	**Model I**	**Model II**	**Model III**	**Model I**	**Model II**	**Model III**
*(I) Socio-demographic variables*						
Age	-.07^*^	.05	.05	-.32^***^	-.17^***^	-.17^***^
Female	.08^*^	.06^*^	.06^*^	-.07^*^	-.10^***^	-.10^***^
Low income	-.02	.01	.01	-.10^***^	.07^**^	-.07^**^
Low education	-.08^**^	-.04	-.05	-.13^***^	-.10^***^	-.11^***^
Single/widow	-.09^**^	-.08^**^	-.09^**^	-.04	-.02	-.03
*(II) APQ dimensions*						
Timeline chronic		.02	.08		-.11^*^	-.00
Timeline cyclical		-.22^**^	-.37^***^		-.20^**^	-.30^**^
Consequence positive		.22^***^	.19^***^		.06	.03
Consequence negative		-.33^***^	-.31^***^		-.40^***^	-.44^***^
Emotional representations		-.03	.11		.13^*^	.20^*^
Control positive		.19^***^	.23^***^		.10^**^	.11^***^
Control negative		-.12^*^	-.08		-.06	-.05
*R* ^2^	.02	.31	.34	.16	.33	.36

*Note.* ^*^ *p* ≤ .05; ^**^ *p* ≤ .01; ^***^ *p* ≤ .001. Standardized coefficients are reported. Coefficients of the APQ and APQ-S are displayed in Model II and III, respectively.

Socio-demographic variables explained 16% of the variance in physical functioning scores (see physical functioning results in Table 4). Including the APQ dimensions increased the explained variance in physical functioning to 33%. Of the APQ dimensions, only consequence positive and control negative were not significantly related to physical functioning. Again, positive relations were found between control positive and physical functioning. Attributing more negative consequences to aging was most strongly related to a lower physical functioning score, followed by having a cyclical awareness and having a chronic awareness of aging.

### APQ-S development and evaluation

#### Item reduction analysis

Areas of localized strain were used to guide item reduction. As aforementioned, several error covariances were found. These error covariances corresponded to two pairs of items on the control positive dimension (item 10 and 11; and item 14 and 15) and two pairs of items comprising timeline cyclical (item 27 and 28; and item 30 and 32). The large error covariances between item 10 and 11 (MI = 176.73, EPC = .39), 27 and 28 (MI = 112.99, EPC = .24), and 30 and 32 (MI = 131.04, EPC = .27) could most likely be attributed to their similarity in wording and/or a theoretical overlap (see Table 1 for the list of items). Consequently, items 11, 28, and 32 were excluded, as they had lower standardized factor loadings and also accounted for more strain within the model. No obvious reason could be provided for the large error covariance between item 14 (‘As I get older there is much I can do to maintain my independence’) and 15 (‘Whether getting older has positive sides to it depends on me’) (MI = 233.68, EPC = .27). Yet, excluding item 11 from the model substantially decreased the error covariance among item 14 and 15 to the point of being non-problematic. Hence, both items were initially kept in the model (item 14 was excluded in a later stage, due to its low standardized factor loading).

Furthermore, modification indices revealed several cross-loadings. Most strongly, item 5 (‘I feel my age in everything that I do’) cross-loaded on the consequence negative dimension (MI = 81.64, EPC = .66). This is not surprising, since the question can be interpreted in terms of discomfort associated with physical decline as a consequence of old age. Another item that was excluded due to cross-loadings was item 24 (‘I have no control over the effects which getting older has on my social life’), which loaded on all but the consequence positive dimension (MI = 18.41 – 56.24, EPC = .18 – .35).

Following the pre-set criteria, seven additional items were excluded, reducing the number of indicators comprising each latent factor to a minimum of three. As the consequence positive dimension already consists of three items, this dimension was kept intact. After item reduction, model fit statistics of the 21-item APQ-S indicated appropriate goodness of fit: *χ*^2^(168) = 589.80, *p* < .001; RMSEA = .05; SRMR = .04; CFI = .94.

#### APQ comparison, reliability, and validity

Inspection of subscale means revealed no large discrepancies between the APQ and APQ-S (see Table 3). The conceptual overlap of both measures is further supported by strong correlations between the APQ and the APQ-S dimensions (*r* = .94 – 1.00).

APQ-S dimensions had a slightly worse internal consistency than their respective APQ dimensions. Yet, most Cronbach’s alphas were above the cut-off point set by George and Mallery [31], indicating acceptable scale reliability (i.e., α ≥ .70). Only the control positive dimension had an internal consistency score just below .70 (α = .69).

While inter-factor correlations were generally weaker for the APQ-S dimensions, correlations remained largely comparable (see Table 3). Also APQ-S regression coefficients were largely similar to the coefficients found when regressing APQ dimensions on wellbeing and physical functioning (see APQ model II and APQ-S model III in Table 4). Only the APQ-S dimensions control negative and timeline chronic lost their significance in explaining wellbeing and physical functioning scores, respectively. Compared to the APQ, APQ-S dimensions explained slightly more variance in wellbeing (Δ *R*^2^ = .03) and physical functioning scores (Δ *R*^2^ = .03).

#### Group differences

As can be expected, older age was significantly correlated with a more chronic and cyclical perception of, attributing more negative consequences to, and harboring more negative emotions towards aging (see Table 5). Furthermore, older age was correlated with perceiving less control over negative events.

**Table 5:** **Correlations between or subgroup means and Cronbach’s alphas for the APQ-S dimensions and regression variables**

	**Timeline chronic**	**Timeline cyclical**	**Consequence positive**	**Consequence negative**	**Emotional representations**	**Control positive**	**Control negative**
*Wellbeing*	-.16^***^	-.36^***^	.21^***^	-.33^***^	-.29^***^	.27^***^	.10^***^
*Physical functioning*	-.34^***^	-.40^***^	-.01	-.49^***^	-.29^***^	.12^***^	.25^***^
*Age*	.31^***^	.18^***^	.02	.32^***^	.09^**^	-.04	-.22^***^
*Gender*							
Female	3.28 (.92)^**^	2.87 (.90)	3.45 (.74)^**^	3.39 (.85)	2.49 (.87)	3.70 (.71)	2.37 (.76)^***^
Male	3.44 (.91)^**^	2.81 (.86)	3.33 (.78)^**^	3.37 (.81)	2.41 (.83)	3.73 (.69)	2.51 (.79)^***^
α_Female_	.81	.77	.74	.80	.79	.70	.76
α_Male_	.81	.74	.75	.77	.78	.68	.75
*Income*							
≤ €1000	3.47 (.93)	2.94 (.88)	3.41 (.79)	3.48 (.84)	2.64 (.97)^*^	3.62 (.71)	2.42 (.84)
> €1000	3.34 (.92)	2.83 (.88)	3.40 (.76)	3.37 (.83)	2.44 (.84)^*^	3.72 (.70)	2.43 (.77)
α_≤ €1000_	.79	.72	.70	.79	.81	.71	.77
α_> €1000_	.81	.76	.75	.79	.79	.69	.76
*Education*							
≤ Elementary	3.45 (.94)^**^	2.89 (.93)	3.36 (.81)	3.45 (.86)^*^	2.58 (.93)^***^	3.68 (.78)	2.28 (.78)^***^
> Elementary	3.30 (.90)^**^	2.82 (.86)	3.42 (.73)	3.35 (.82)^*^	2.40 (.81)^***^	3.73 (.66)	2.50 (.76)^***^
α_≤ Elementary_	.81	.75	.72	.77	.79	.77	.76
α_> Elementary_	.81	.76	.75	.80	.79	.64	.75
*Marital status*							
Single/widow	3.38 (.94)	2.91 (.91)^***^	3.43 (.76)	3.42 (.86)^*^	2.50 (.89)^*^	3.72 (.73)	2.36 (.77)^***^
Married/living together	3.30 (.88)	2.74 (.83)^***^	3.36 (.75)	3.32 (.78)^*^	2.39 (.80)^*^	3.69 (.67)	2.53 (.77)^***^
α_Single/widow_	.82	.77	.73	.80	.79	.71	.74
α_Married/living together_	.79	.73	.76	.77	.78	.66	.77
*Ethnicity*							
Non-Dutch	3.33 (.97)	2.84 (.97)	3.41 (.86)	3.25 (.92)^*^	2.48 (.86)	3.70 (.84)	2.57 (.88)^*^
Dutch	3.35 (.91)	2.84 (.87)	3.40 (.74)	3.40 (.82)^*^	2.45 (.85)	3.71 (.68)	2.40 (.75)^*^
α_Non-Dutch_	.82	.83	.81	.81	.79	.75	.79
α_Dutch_	.81	.74	.73	.79	.79	.67	.75

*Note.* ^*^ *p* ≤ .05; ^**^ *p* ≤ .01; ^***^ *p* ≤ .001. Subgroup differences were calculated using independent sample *t*-tests.

On average, male and lower educated respondents reported a more chronic awareness of aging. A cyclical awareness was only stronger among single/widowed respondents. While female respondents attributed more positive consequences to the aging process, respondents who were single/widowed, low educated, or Dutch attributed more negative consequences to aging. Those who were single/widowed, low educated, and had a lower income related more negative emotions to the aging process. Finally, non-Dutch elders perceived more control over negative events, as did those who were married or living together, or had a higher educational level. Finally, as can be seen in Table 5, similar Cronbach’s alphas were found for most dimensions across subgroups.

#### B-APQ comparison

Items comprising the dimensions of the APQ-S and B-APQ were compared in order to better assess the broad differences between the instruments. The B-APQ is a 5-factor model, including the dimensions timeline chronic (items 3, 4, and 5), consequence positive (items 6, 7, and 8), control positive (items 10, 11, and 12), emotional representations (items 9, 26, and 29), and the combined consequence and control negative dimension (items 17, 19, 20, 21, and 24; control negative dimensions are not reverse coded) [16].

In addition to the absence of the timeline cyclical dimension and the combined consequence and control negative dimension, several other notable differences can be found. First, items 10 (‘The quality of my social life in later years depends on me’) and 11 (‘The quality of my relationships with others in later life depends on me’) are included in the control positive dimension, while items 14 and 15 are deleted. However, in the APQ-S it was specifically chosen to exclude item 11 due to a large theoretical overlap with item 10 and to keep item 15. Second, item 5 (‘I feel my age in everything that I do’), which was deleted in the APQ-S due to its high cross-loading on the consequence negative dimension, is still present in the B-APQ. Also item 24, which had cross-loadings on all but one dimension, is still present in the model.

To examine the model fit of the B-APQ, an additional CFA was conducted. The resulting model had an acceptable model fit: *χ*^2^ (109) = 417.33, *p* < .001; RMSEA = .05; SRMR = .05; CFI = .93. Model fit was significantly worse than the APQ-S (Δ *χ*^2^ (59) = 120.88, *p* < .001).

Following, Cronbach’s alphas were calculated for each dimension of the B-APQ and compared to the Cronbach’s alphas of the APQ-S. As can be seen in Table 6, each dimension had an acceptable internal consistency (α ≥ .70). The combined consequence and control negative dimension had a slightly lower Cronbach’s alpha than its respective separate dimensions in the APQ-S (see Table 3). The control positive dimension of the APQ-S, on the other hand, had a relatively low internal consistency compared to its B-APQ duplicate (α = .69 instead of α = .79).

**Table 6:** **Inter-factor and inter-instrument correlations for the B-APQ**

**APQ Dimensions**	**1.**	**2.**	**3.**	**4.**	**5.**	**M (SD)**	**α**
*1. Chronic*	-					3.17 (.94)	.81
*2. Consequence positive*	.19^***^	-				3.40 (.76)	.74
*3. Consequence & control negative*	.23^***^	.08^**^	-			3.02 (.41)	.72
*4. Emotional representations*	.45^***^	.12^***^	.25^***^	-		2.57 (.84)	.70
*5. Control positive*	.05	.24^***^	.04	-.01	-	3.74 (.76)	.79
*B-APQ & APQ-S*	.93^***^	1.00^***^	.55^***1^	.92^***^	.91^***^	-	-
			-.01^2^				

*Note.* ^**^p ≤ .01; ^***^p ≤ .001. ^1^Inter-instrument correlation between the combined consequence and control negative dimension and the consequence negative dimension of the APQ-S. ^2^Inter-instrument correlation between the combined consequence and control negative dimension and the control negative dimension of the APQ-S.

Finally, inter-factor and inter-instrument correlations were calculated. As can be seen in Table 6, inter-factor correlations were largely comparable. Inter-instrument correlations, however, revealed that while the combined consequence and control negative dimension had a strong correlation with the consequence negative dimension of the APQ-S, its correlation with the control negative dimension of the APQ-S was much lower. Hence, the combined consequence and control negative dimension is a better representation of the consequence negative than the control negative dimension.

## Discussion

In an aging society, the availability of valid and reliable instruments measuring particular aspects of the aging process has become of great importance. With its focus on the subjective experience of aging, the APQ can provide interesting insight into the influence of perceptions of aging on various aspects of life. The objective of the current study was to (a) examine the psychometric properties of the APQ in a sample of community-dwelling elders in the Netherlands and (b) shorten the questionnaire while retaining its validity, reliability, and its 7-factor structure.

This study provided support for the use of the self-regulation model [9] to the context of aging [8]. Yet, while each item in the APQ loaded significantly onto its corresponding latent dimensions, CFA results suggested that model respecification was needed to warrant further use among the Dutch elderly population. Each APQ dimension was found to have acceptable reliability. Furthermore, inter-factor correlations broadly supported the construct validity of the APQ. Only the consequence positive scale behaved in an unexpected manner, as will be discussed in a later section. In general, expected relations were found between the APQ dimensions and wellbeing and physical functioning scores, providing additional evidence for construct validity and further underscoring the significance of aging perceptions for various aspects of health and quality of life at old age [2-8].

Item reduction resulted in a 21-item version of the APQ. As the areas of strain were addressed during item reduction, the resulting APQ-S had a notably better model fit than the original APQ. Furthermore, reliability and construct validity were preserved. There was, however, a slight decrease in Cronbach’s alpha for the APQ-S. Yet, as a reduction in the number of items comprising a scale may artificially deflate the Cronbach’s alpha [28], this decrease is not alarming. Furthermore, subscale reliability remained adequate after item reduction, with only the control positive dimension scoring slightly below the preset cut-off point. Additional tests revealed that the APQ-S can reliably be used among different subgroups. Furthermore, expected group differences were found for the APQ-S, further supporting its construct validity. For example, those who were single/widowed, lower educated, or had an income equal to or lower than € 1000 attributed more negative emotions to the aging process. Compared to the B-APQ, the APQ-S had a slightly better model fit. Yet, this could be expected, since model respecification, which resulted in the APQ-S, was conducted on the current sample. The internal consistency of the control positive subscale, however, was notably higher for the B-APQ. Inter-instrument correlations revealed that control negative lost a large part of its relevance in the combined consequence and control negative dimension, as visible in the weak correlation between control negative and the combined dimension and the strong correlation for consequence negative.

### Consequence positive inter-factor correlations

A finding that has to be discussed in more detail is the inter-factor correlations for the consequence positive scale. In the current study, those who more strongly defined aging in terms of becoming wiser, more appreciative, and developed also perceived to be less in control over negative experiences related to aging and generated more negative emotional responses and attributed more negative consequences to aging. Furthermore, they were more chronically aware of and experienced more variations in their aging process.

While the negative correlation between consequence positive and control negative has also been found by Sexton and colleagues [16], the other contradicting relations are unique to this study and may point to a cross-country instability in the construct. It could be that Dutch elderly who reflect more on their aging tend to do so in a way that highlights their personal development. Yet, a heightened awareness of aging may also imply being more aware of the negative consequences of aging, e.g., becoming more dependent. These negative consequences may especially be likely in the Netherlands, as increasing retrenchments in social security brought forth large reforms in elderly care, transferring the burden of cost from the state to the individual and moving from formal towards informal care [32]. These reforms have had a large impact on the lives of Dutch elders, as care subsidies have shrunk and, due to stricter regulations, fewer people have become eligible for formal care. As a consequence, Dutch elders are increasingly charged with the costs and responsibility of care and have become more dependent upon relatives for help. Elderly people may find it hard to cope with these rapid changes and may worry about the financial costs and/or the effect of ‘informalizing’ care on the relationship with their (‘already busy and financially strained’) relatives. In a study among 530 disabled and elderly Dutch citizens, it was found that the majority of respondents experienced or anticipated problems due to the recent reforms in long-term care. Especially their increased dependence upon informal networks was seen as a problem, as it would threaten their autonomy and reputation as well as burden their family, a source of support on which most did not want to structurally rely [33]. Hence, while in the Netherlands aging may be defined in positive terms on a personal level, on a broader level, aging likely implies an increased financial and filial burden. Future research is needed to more closely inspect these inter-relations.

### APQ-S or B-APQ?

The question remains whether one should use the APQ-S or the B-APQ as an alternative to the lengthy APQ. With its even smaller set of items and dimensions, use of the B-APQ could facilitate data gathering and analyses to a greater extent. However, several points of concern have to be addressed.

First, by combining the consequence and control negative dimension, it is not possible to discern their independent influence on outcome measures. Subsequently, it becomes difficult to provide a clear interpretation of significant relations. Furthermore, it could be questioned whether the favorable fit statistics of the combined consequence and control negative dimension are due to a theoretical overlap of these dimensions – both dimensions were argued to capture loss of control and physical decline [16] – or to a method bias originating from people’s tendency to answer in a similar manner to negatively phrased items [28,34].

Second, interesting information could be lost by deleting the timeline cyclical dimension. The decision to exclude timeline cyclical was based on the rationale that this dimension is less relevant to aging perceptions, as the primary purpose of the timeline cyclical dimension was to capture representations of cyclical or episodic illness, such as allergies [8,16]. Yet, it could be argued that this dimension is in fact important to assess aging perceptions, since one’s awareness of aging could episodically increase or decrease as a consequence of life changes. To illustrate, an elderly person who otherwise would not describe himself as old might become aware of his old age when he breaks his hip and becomes immobile for a period of time, when he experiences a momentary increase in chronic or age-related disease symptomatology, or when a close relative, friend, or neighbor dies. The importance of the timeline cyclical dimension is further underscored by its significant and relatively strong relation with wellbeing and physical functioning in this study.

Finally, the set of items comprising the control positive dimension of the B-APQ could be reason for concern. In the control positive dimension of the B-APQ both item 10 (‘The quality of my social life in later years depends on me’) and 11 (‘The quality of my relationships with others in later life depends on me’) are kept. Yet, these items have a large theoretical overlap and largely represent the same factor. Thereby, inclusion of both items may shift the focus of the concept from a more general control over positive experiences to a more specific control over social experiences. By excluding one of the two items and retaining item 15, the APQ-S provides a more holistic representation of the concept. Even though this decision compromised the internal consistency of the scale, the theoretical assumptions underlying the concept are better represented.

It is not a clear-cut decision whether one should use the APQ-S or B-APQ for future research. While the B-APQ is more concise with its smaller number of items and dimensions, several concerns have been raised. The final decision to either use the APQ-S or B-APQ should therefore be based on the weight placed upon above mentioned concerns and the theoretical and practical implications of using the original 7-dimensional structure, i.e., the APQ-S, or the 5-dimensional structure of the B-APQ for future study design.

### Limitations

Several limitations should be taken into account when interpreting our study findings. First, as the APQ-S was developed following respecification conducted on the current sample, it was not possible to draw any conclusions on the model fit of the APQ-S compared to the B-APQ. To better assess and compare the model fit of both APQ versions, additional validation studies have to be conducted using different study samples. Second, with a response rate of 46%, the study sample may have introduced non-response bias. We therefore compared the study sample (*n* = 1280) to the original sample (*n* = 2798). No difference in age was found. We did however find a difference in gender (57.6% females in the study sample versus 62.3% females in the original sample) and ethnicity (15.2% non-Dutch in the study sample versus 20.1% non-Dutch in the original sample). While these differences appear to be small, they might point towards a selective non-response and thus a bias in the results. For ethnicity, the lower response rate could be expected, since ethnic minority groups generally are less willing or able to participate in surveys [35]. Deploying data collection strategies that are more tailored to the target group may be helpful to reduce this selective non-response. For instance, increasing the number of contact attempts has been suggested to reduce non-response among immigrants [35]. Finally, as this study made use of cross-sectional data, causal inferences cannot be made. While negative perceptions of aging may be maladaptive to the physical functioning and wellbeing of elderly people, it is also highly likely that a more negative outlook on life or physical discomforts influence how people perceive their aging process. Underscoring the latter, indicators of wellbeing and physical functioning have been suggested as prerequisites for successful adaption to the aging process [36,37]. Hence, longitudinal data are needed to better assess the directionality of these relationships. Furthermore, longitudinal data would also allow for testing predictive validity.

## Conclusion

Since perceptions of aging have been found to be of key importance to the health and wellbeing of elderly people, the APQ may prove to be a valuable instrument to geriatric research. The development and validation of the Dutch version of the APQ opens the door for detailed examination of aging perceptions in the Netherlands and other Dutch-speaking countries. Furthermore, the use of the APQ-S or B-APQ in international geriatric studies, as an alternative to the lengthier APQ, may be advisable, as it alleviates part of the response burden among elderly participants and, in turn, might increase study participation. The choice between the APQ-S and the B-APQ should be based on theoretical and practical considerations concerning the dimensional structure most suitable for the study.

## Additional file

Additional file 1:
**Dutch-language APQ.** Dutch-language Aging Perceptions Questionnaire, perceptions of aging scale.
