# Supplementary material for: Validation of the Dutch Aging Perceptions Questionnaire and development of a short version
Source: Health Qual Life Outcomes. 2015 May 12;13:54. doi: 10.1186/s12955-015-0248-y (PMC4426604; doi:10.1186/s12955-015-0248-y)
Supplement: Additional file 1: — Dutch-language APQ. Dutch-language Aging Perceptions Questionnaire, perceptions of aging scale. [file 12955_2015_248_MOESM1_ESM.docx]

**Additional file:** Aging Perceptions Questionnaire (APQ) perceptions of aging scale (English original [8] and Dutch translation).

| 1. I am conscious of getting older all of the time | Ik realiseer me dat ik steeds ouder word |
| --- | --- |
| 1. I am always aware of my age | Ik ben me altijd bewust van mijn leeftijd |
| 1. I always classify myself as old | Ik zie mezelf altijd als een ouder iemand |
| 1. I am always aware of the fact that I am getting older | Ik ben me altijd bewust van het feit dat ik ouder word |
| 1. I feel my age in everything that I do | Ik voel mijn leeftijd bij alles wat ik doe |
| 6. As I get older I get wiser | Naarmate ik ouder word, word ik wijzer |
| 7. As I get older I continue to grow as a person | Naarmate ik ouder word, neemt mijn persoonlijke groei toe |
| 8. As I get older I appreciate things more | Naarmate ik ouder word, waardeer ik dingen steeds meer |
| 9. I get depressed when I think about how  ageing might affect the things that I can do | Ik word somber als ik bedenk wat het ouder worden betekent voor wat ik nog kan doen |
| 10. The quality of my social life in later years  depends on me | De kwaliteit van mijn sociale leven op latere leeftijd hangt af van mijzelf |
| 11. The quality of my relationships with others  in later life depends on me | De kwaliteit van mijn relaties met anderen op latere leeftijd hangt af van mijzelf |
| 12. Whether I continue living life to the full  depends on me | Of ik nog altijd een volwaardig leven kan leiden hangt af van mijzelf |
| 13. I get depressed when I think about the  effect that getting older might have on my social life | Als ik bedenk wat het ouder worden betekent voor mijn sociale leven, word ik somber |
| 14. As I get older there is much I can do to  maintain my independence | Er is veel dat ik kan doen om zelfstandig te blijven, ook al word ik ouder |
| 15. Whether getting older has positive sides to  it depends on me | Of het ouder worden positieve kanten heeft hangt af van mijzelf |
| 16. Getting older restricts the things that I can  do | Het ouder worden beperkt mijn mogelijkheden |
| 17. Getting older makes me less independent | Het ouder worden maakt me minder onafhankelijk |
| 18. Getting older makes everything a lot harder  for me | Het ouder worden maakt alles veel moeilijker voor mij |
| 19. As I get older I can take part in fewer  activities | Naarmate ik ouder word, kan ik aan minder activiteiten deelnemen |
| 20. As I get older I do not cope as well with  problems that arise | Naarmate ik ouder word, kan ik minder goed omgaan met nieuwe problemen |
| 21. Slowing down with age is not something I  can control | Trager worden op hogere leeftijd is niet iets dat ik zelf in de hand heb |
| 22. How mobile I am in later life is not up to me | Het ligt niet aan mij hoe mobiel ik ben op latere leeftijd |
| 23. I have no control over whether I lose vitality  or zest for life as I age | Ik kan er zelf niets aan doen als ik later minder vitaal word of mijn levenslust verlies |
| 24. I have no control over the effects which  getting older has on my social life | Ik kan zelf niets doen aan het effect van het ouder worden op mijn sociale leven |
| 25. I get depressed when I think about getting  older | Denken aan het ouder worden maakt me somber |
| 26. I worry about the effects that getting older  may have on my relationships with others | Ik maak me zorgen over het mogelijke effect van het ouder worden op mijn relaties met anderen |
| 27. I go through cycles in which my experience  of ageing gets better and worse | Ik zit in een cyclus waarin ik afwisselend betere en slechtere ervaringen heb met het ouder worden |
| 28. My awareness of getting older comes and  goes in cycles | Dat ik me bewust ben van het ouder worden komt en gaat bij mij in golven |
| 29. I feel angry when I think about getting older | Denken aan het ouder worden maakt me boos |
| 30. I go through phases of feeling old | Er zijn tijden dat ik me oud voel |
| 31. My awareness of getting older changes a  great deal from day to day | Van dag tot dag heb ik een ander besef van het ouder worden |
| 32. I go through phases of viewing myself as  being old | Er zijn tijden dat ik mijzelf oud vind |
